# The Wnt/β-catenin signaling pathway in colorectal cancer: mechanism and intervention of traditional Chinese medicine and chemical compound

**DOI:** 10.3389/fphar.2025.1560714

**Published:** 2025-04-10

**Authors:** Sha Zeng, Juan Wang, Zhengrong Shi, Hui Zhao, Jingxing Gao, Jinxiu Li

**Affiliations:** ^1^ Chengdu Integrated TCM and Western Medicine Hospital, Department of Traditional Chinese Medicine Pharmacy, Chengdu, China; ^2^ Henan University of Traditional Chinese Medicine, Department of pharmacology, Zhengzhou, Henan, China

**Keywords:** colorectal cancer (CRC), the Wnt/β-catenin signaling pathway, mechanism, traditional Chinese medicine (TCM), chemical compound

## Abstract

Colorectal cancer (CRC) is globally recognized as the third most frequently diagnosed malignancy and the second leading cause of cancer-related mortality. The etiology of CRC is multifactorial, arising from a complex interplay of genetic alterations, environmental exposures, and age-related physiological changes. Among the numerous signaling pathways that regulate cellular homeostasis, the Wnt/β-catenin signaling pathway not only plays a critical role in embryonic development and cell proliferation but also contributes to the initiation and progression of various malignancies, including CRC. Dysregulation of the Wnt/β-catenin signaling pathway is a hallmark of CRC, playing a pivotal role in regulating chemoresistance and driving invasive and metastatic behaviors. Traditional Chinese Medicine (TCM) is characterized by its multi-target and multi-pathway mechanisms. Extensive studies have demonstrated that TCM can inhibit the activity of CRC cells by targeting the Wnt/β-catenin signaling pathway and significantly alleviate symptoms in CRC animal models, demonstrating its potential therapeutic value for the treatment of CRC. This review primarily focuses on the literature published in the past 5 years, retrieved from databases such as PubMed, Web of Science, Scopus, MEDLINE, and Springer, concerning the targeting of the Wnt/β-catenin signaling pathway for the treatment of CRC. It highlights the research progress on TCM monomers (e.g., myricetin, genistein, baicalein), TCM formulations (e.g., Pai-Nong-San (PNS), Jian-Du-Xiao-Sheng Yin (JXY), Zuo-Jin-Wan (ZJW)), and small-molecule inhibitors (e.g., PCDHGA9, Cetuximab, PTK7). Furthermore, the experimental results and conclusions from these studies are thoroughly analyzed and discussed. Through a comprehensive review of the literature, we conclude that TCM exhibits multi-level, multi-target, and multi-faceted effects in the prevention and treatment of CRC. In-depth research into the mechanisms by which TCM targets the Wnt/β-catenin signaling pathway to prevent and treat CRC may provide novel insights into exploring the pathogenesis of CRC and developing new therapeutic agents for CRC.

## 1 Introduction

Colorectal cancer (CRC) is recognized as a clonal disease (Smith et al., 2021). Epidemiological studies indicate that the progression of most CRC cases is associated with geography, gender, and age (Sullivan et al., 2022). In Western countries, North America, Australia and New Zealand are high-incidence areas while West Africa and South Asia have lower incidence rates of CRC (Fitzmaurice et al., 2017; Siegel et al., 2023). The incidence and mortality rates of CRC in China are increasing yearly. According to the 2022 cancer statistics in China (Han et al., 2024), the incidence of CRC in China currently ranks 63rd in the world. The number of newly diagnosed cases of CRC is 517,100, which represents 26.8% of the global incidence of CRC. The mortality rate ranks 73rd worldwide, with 240,000 newly reported CRC-related deaths, representing 26.5% of global CRC deaths. By 2030, the global CRC burden is expected to increase by 60% with 2.2 million new cases and 1.1 million deaths (Mauri et al., 2025).

Currently, the majority of newly diagnosed CRC cases are classified as sporadic forms, lacking a family history or inherited genomic alterations, and these account for approximately 60%–65% of all CRC cases. The pathogenesis of sporadic CRC is predominantly mediated by acquired somatic genetic mutations and epigenetic alterations, which are induced by modifiable risk factors. In contrast, approximately 35%–40% of CRC cases demonstrate a predisposition associated with heritable factors (Mármol et al., 2017). These factors encompass a family history of CRC in the absence of a distinct genetic predisposition, inherited cancer syndromes (e.g., Lynch syndrome), low-penetrance genetic variants, and other unidentified inherited genomic abnormalities. Importantly, environmental factors significantly contribute to the carcinogenic process across all CRC cases. Even in patients with a family history of CRC, acquired genomic alterations often remain the primary driver of disease progression (Rawla et al., 2019; Valle et al., 2019). The pathogenesis of CRC represents a highly complex, protracted, and multistep process. Conventional CRC typically originates from morphological changes in colonic epithelial cells, which undergo uncontrolled proliferation to form benign polyps. Over time, these polyps evolve into highly dysplastic advanced adenomas, leading to the loss of epithelial structure and function, and ultimately progressing to invasive carcinomas (Li et al., 2024; Angarita et al., 2018). This stepwise progression underscores the complex interplay of genetic, epigenetic, and environmental factors in CRC development.

The Wnt/β-catenin signaling pathway is pivotal in the morphogenesis of stem cells within diverse body tissues and their subsequent regeneration processes. Aberrant activation of this pathway is closely associated with a spectrum of growth-related disorders and malignancies. Notably, it serves as a linchpin in propelling the initiation and progression of CRC (Bian et al., 2020). The Wnt/β-catenin signaling pathway mainly contains several target proteins, including low-density lipoprotein receptor-related protein 5/6 (LRP5/6), glycogen synthase kinase 3 beta (GSK3β), adenomatous polyposis coli (APC), Axin and β-catenin. Genetic variations in these components of the Wnt/β-catenin signaling pathway may activate the β-catenin protein, thereby increasing the proliferation of CRC cells (Aghabozorgi et al., 2020). Currently, the Wnt/β-catenin signaling pathway has been identified as a significant factor contributing to cancer deterioration. It influences tumor development by meticulously controlling key processes, such as the regulated progression of the tumor cell cycle, the elaborate process of angiogenesis, and the pronounced invasive capabilities of tumor cells, including invasion and metastasis (Yu et al., 2021). Consequently, a thorough investigation of the Wnt/β-catenin signaling pathway is of paramount importance for the medical management, postoperative recovery, and recurrence prevention in CRC.

Although previous studies have confirmed that traditional Chinese medicine (TCM) can intervene in the progression of CRC at different pathological stages by regulating the Wnt/β-catenin signaling pathway (Chang et al., 2021), the precise molecular mechanisms underlying the therapeutic efficacy of diverse bioactive metabolites in TCM remain to be comprehensively elucidated. While this review exhibits certain parallels with the aforementioned study, it differentiates itself by offering a thorough and current explanation of how the Wnt/β-catenin signaling pathway plays a role in the development of CRC, backed by the most recent scientific research. Furthermore, this review presents a comprehensive and systematic analysis of the molecular targets and mechanistic foundations associated with TCM monomers, TCM formulations, and small-molecule inhibitors that modulate the Wnt/β-catenin signaling pathway, integrating the latest advancements in the field. This systematic and mechanistic focus constitutes the most significant distinction between the two works.

## 2 Overview of the Wnt/β-catenin signaling pathway

The Wnt/β-catenin signaling pathway is structured into four segments: extracellular signaling, membrane-bound fragment, cytoplasmic fragment, and nuclear fractions (Nusse and Clevers, 2017). Extracellular signals primarily include the Wnt family of secreted proteins. The membrane-bound fragment is mainly composed of Frizzled (Fzd) and LRP6 proteins. In the cytoplasmic fraction, key components include Disheveled (DVL), GSK3β, Axin, Casein kinase 1 (CK1), APC, and β-catenin proteins. The nuclear fraction comprises β-catenin translocated to the nucleus, T cell factor (TCF), and lymphoid enhancer factor (LEF).

Wnt ligands, comprising 19 glycoproteins, function through paracrine or autocrine signaling. As a key nuclear effector of the Wnt/β-catenin pathway, the β-catenin protein’s primary structure consists of an amino-terminal region of approximately 130 amino acids and a central region of 550 amino acids. Additionally, it includes a carboxyl-terminal region of about 100 amino acids, along with 12 imperfect repeat sequences, each containing 42 amino acids, known as armadillo (arm) repeats (Wang et al., 2021a). These repeats form a long, supercoiled groove with a positive charge (Hayat et al., 2022). In the Wnt/β-catenin signaling pathway, GSK3β regulates the phosphorylation of β-catenin, thereby promoting its proteasome-mediated degradation and playing a critical role in controlling the transcriptional activity of β-catenin (Dema et al., 2016; Fan et al., 2025). APC, a tumor suppressor, is instrumental in regulating the growth of normal cells and inhibiting the proliferation of abnormal cells in the colorectal region. Consequently, APC is considered a fundamental etiological factor in the progression of CRC (Lesko et al., 2015). Mutation of APC leads to hyperactivation of the Wnt/β-catenin signaling pathway, resulting in the formation of adenomas in murine intestines (Zhang et al., 2017). This hyperactivation precipitates the constitutive activation of the pathway, which is a key process in the dysregulation of cell growth, differentiation, and chromosomal instability in human colorectal adenocarcinoma (LaBella et al., 2024).

**FIGURE 1:**
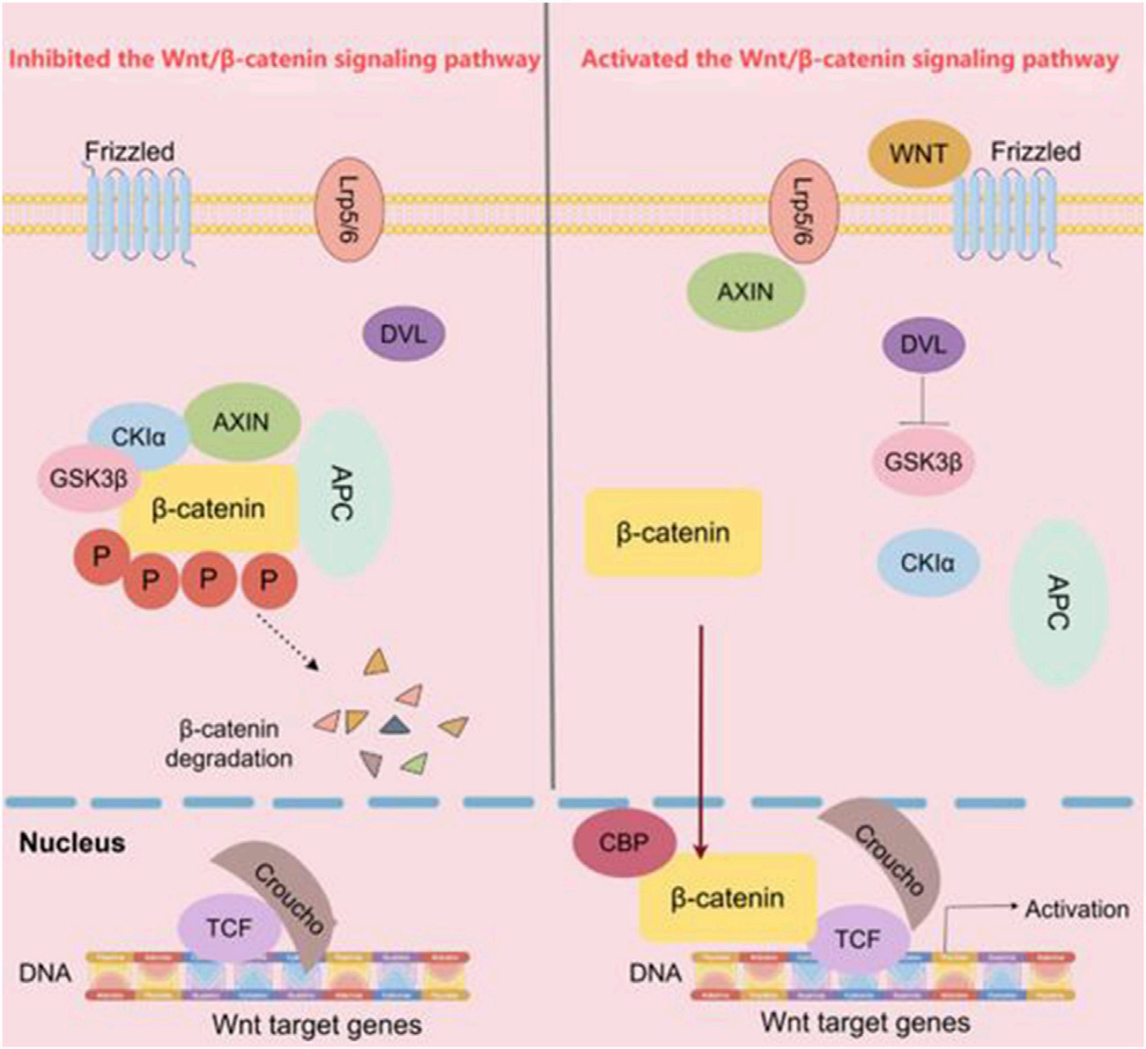
The Wnt/β-catenin signaling pathway. In the classical Wnt/β-catenin pathway, when Wnt ligands are absent, Axin binds to β-catenin, GSK3β, and APC. This allows GSK3β to phosphorylate β-catenin, leading to its ubiquitination and subsequent degradation by the APC. As a result, β-catenin is prevented from entering the nucleus and initiating downstream gene transcription. Conversely, in the presence of Wnt ligands, the Wnt receptor binds to Axin, preventing β-catenin from interacting with Axin, GSK3β, and APC. Consequently, β-catenin accumulates and translocates into the nucleus, where it initiates downstream transcription. (The figure was created by Figdraw, https://www.figdraw.com/static/index.html#/).

When the Wnt/β-catenin signaling pathway is unactive, β-catenin is phosphorylated by a complex consisting of APC, Axin, and GSK3β. This phosphorylation predominantly targets serine and threonine residues at the N-terminus of β-catenin, which promotes the binding of β-catenin to the β-transducin repeat-containing protein (β-TRCP), initiating the subsequent degradation process. The E3 ubiquitin ligases degrades β-catenin, leading to a decrease in the cytoplasmic levels of β-catenin. Consequently, the downstream transcription factors of the Wnt/β-catenin signaling pathway cannot be expressed (Wu et al., 2024). Once the body is stimulated by external factors, the Wnt/β-catenin signaling pathway is activated through the binding of the Wnt protein-ligand to the Fzd. After 7 times of receptor transmembrane, Fzd transmits biological signals to DVL in the cell (Bruguera et al., 2025). A significant portion of DVL is recruited to the plasma membrane. Upon undergoing extensive phosphorylation, activated DVL facilitates the assembly of Fzd and LRP6. This clustering event subsequently enhances the phosphorylation of LRP6, thereby amplifying the signaling cascade. Simultaneously, APC and Axin act as key regulators by preventing GSK3β from phosphorylating β-catenin. Consequently, unphosphorylated β-catenin cannot be recognized by β-TRCP. Due to the lack of recognition, β-catenin degradation is impeded, resulting in its accumulation within the cell and eventual entry into the nucleus. In the nucleus, β-catenin binds to the LEF/TCF4 complex and activates the expression of downstream target genes such as Cyclin D1 and c-Myc (Ge et al., 2020; Song et al., 2015), ultimately leading to the occurrence and development of cancer (Figure 1).

## 3 The Wnt/β-catenin signaling pathway and CRC

### 3.1 Impact of the Wnt/β-catenin signaling pathway on the cell cycle progression of CRC cells

**FIGURE 2:**
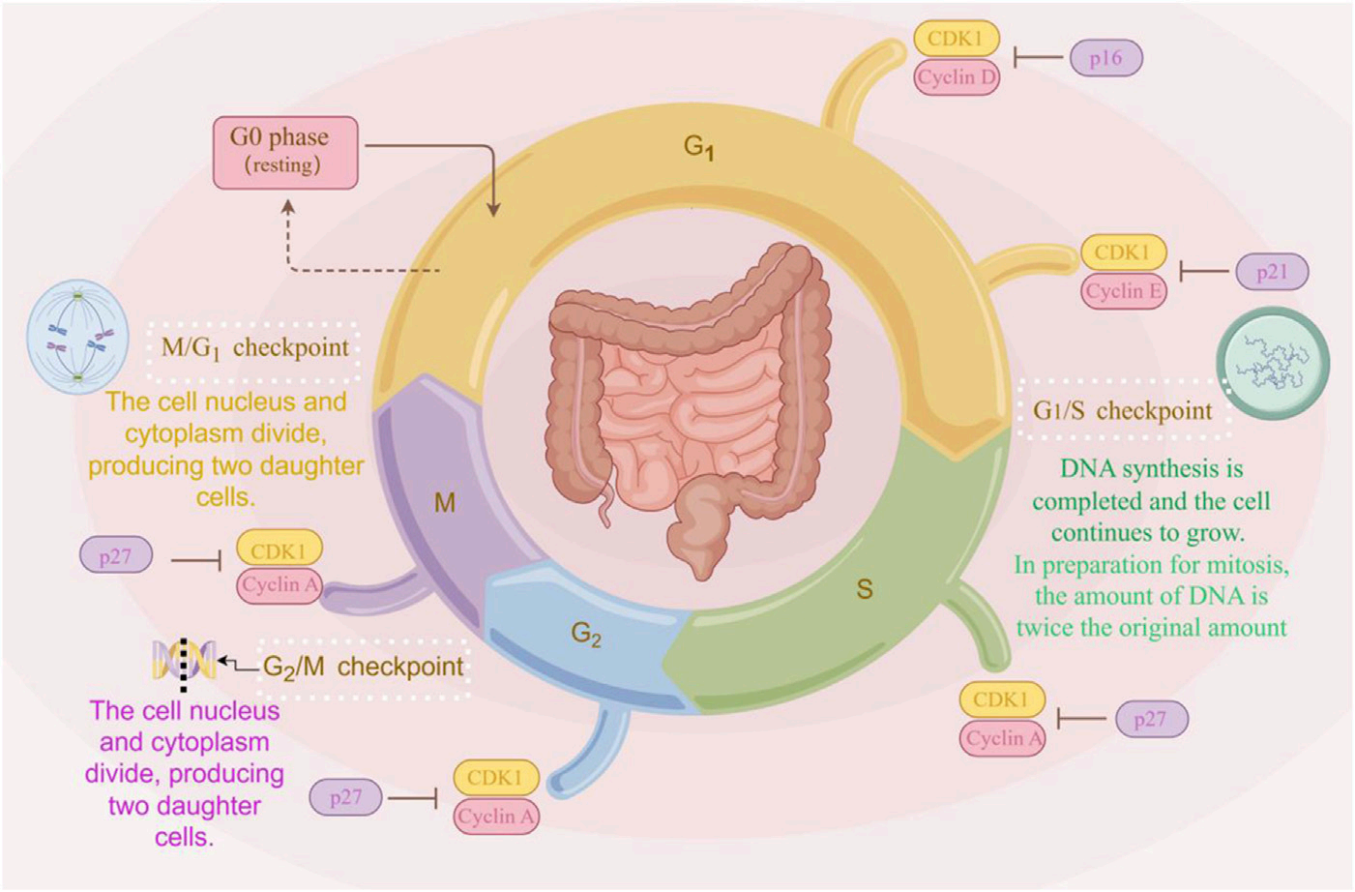
The cell cycle progression. The cell cycle comprises four phases: G1, S, G2, and M. Cells enter the G1 phase after mitosis, during which RNA and protein synthesis occur, but DNA replication does not. The transition from G1 to S phase is marked by the onset of DNA replication. The S phase continues until the entire DNA complement is replicated, increasing from the diploid state (2n) to the tetraploid state (4n). The period from the end of the S phase until mitosis is referred to as the G2 phase, during which cells contain two complete sets of diploid chromosomes. Nuclear volume increases during the S phase as proteins accumulate in parallel with DNA replication. Throughout this process, the chromatin remains condensed, with no visible changes in morphology (The figure was created by Figdraw, https://www.figdraw.com/static/index.html#/).

The cell cycle, which completes the continuation of life by triggering cell division to produce two new cells, is one of the indispensable processes in the life of an organism. During cell proliferation and mitosis in the cell cycle, error-free chromosome segregation is critical (Jamasbi et al., 2022). As shown in Figure 2, the eukaryotic cell cycle is composed of G1 (prophase), S (metaphase), G2 (anaphase), and M (telophase) phases. During the cell cycle, DNA replication, gene transcription, and protein translation all occur in a cell cycle-dependent manner (Wang, 2022). The cell cycle is a critical determinant in the progression of cancer. Aberrant activation of the Wnt/β-catenin signaling pathway contributes to cell proliferation, differentiation, and renewal of cancer stem cells (Nusse et al., 2017). In the majority of CRC patients, overexpression of target genes in the Wnt/β-catenin signaling pathway leads to cell cycle dysregulation, promoting tumor invasion and metastasis (Qi et al., 2015a).

Modulation of the Wnt/β-catenin signaling pathway mediates the anti-proliferative and anti-metastatic effects of emodin in CRC. This is achieved through downregulation of β-catenin transcription factor-7-like-2 (TCF7L2) mRNA and suppression of downstream Wnt/β-catenin signaling target genes, including Cyclin D1, c-Myc, Snail, Vimentin, matrix metalloproteinase 2 (MMP2), and MMP9, in human CRC cells (Pooja et al., 2014). Additionally, Yang et al. (Yang et al., 2021) found that Ginkgolide C (GGC) could downregulate the expression of the Wnt/β-catenin signaling cascade, including MMP2, MMP9, Wnt3a, β-catenin, and the downstream signaling components of β-catenin such as Axin1, p-GSK3β, and β-TRCP, as well as the target genes (c-Myc, Cyclin D1, and survivin) in HT-29 cells. It is indicated that GGC may exert anti-proliferation, anti-invasion, anti-migration, and pro-apoptosis effects on CRC cells by targeting the Wnt/β-catenin signaling pathway.

### 3.2 Effect of the Wnt/β-catenin signaling pathway on angiogenesis in CRC

**FIGURE 3:**
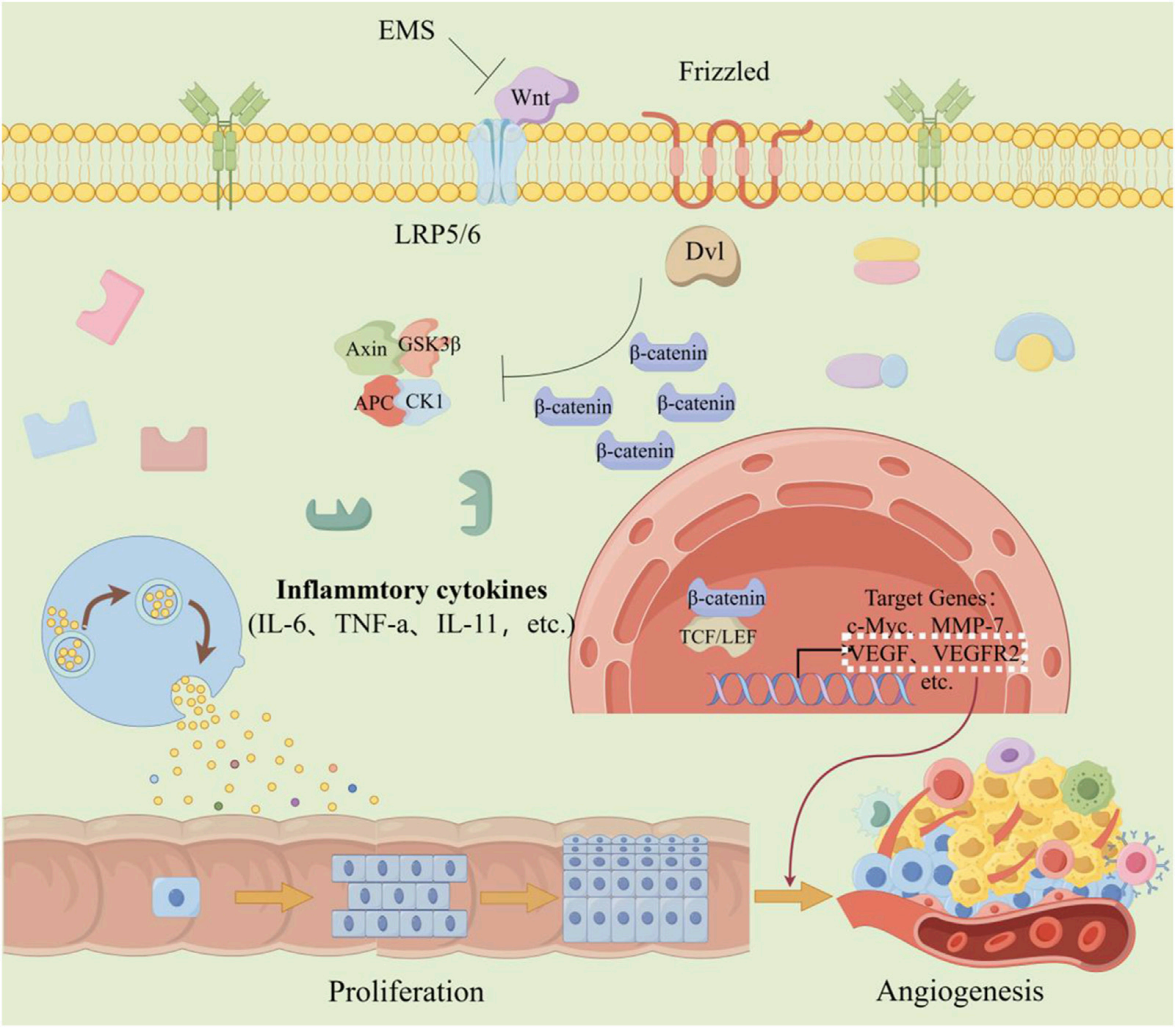
The effect of angiogenesis in CRC. When colorectal cells are chronically stimulated by inflammation, mechanisms that trigger abnormal cell proliferation are activated. Simultaneously, the Wnt/β-catenin signaling pathway is abnormally activated, resulting in increased expression of VEGF and VEGF2-factors associated with angiogenesis. This further promotes the proliferation of cancer cells and the formation of blood vessels. (The figure was created by Figdraw, https://www.figdraw.com/static/index.html#/).

Angiogenesis is a critical process in the progression of tumor growth and metastasis. Not only is it essential for sustaining tumor expansion, but it also serves as a prerequisite for tumor cells to penetrate the vasculature. This process involves the generation of new blood vessels that connect with the existing circulatory system (Razavi et al., 2021). Angiogenesis plays a key role in CRC (Figure 3). The activity of vascular endothelial growth factor (VEGF) family proteins and their receptor, VEGFR, is upregulated through the targeting of multiple signaling pathways, promoting endothelial cell proliferation, migration, differentiation, and an increase in vascular permeability (Lopez et al., 2019). During the early stages of CRC development, levels of VEGF are elevated, and this increase becomes more pronounced in the later (metastatic) stages of CRC (Tacconi et al., 2015).

The classical Wnt/β-catenin signaling pathway is also involved in tumor vasculogenic mimicry (VM) (Kasprzak, 2020). Results showed that the expression of Wnt3a and the nuclear localization of β-catenin were significantly higher in VM-positive cells compared to VM-negative cells. *In vitro* and mouse xenograft model studies have confirmed and explained that the mechanism by which over-regulated Wnt3a participates in this process is to increase the expression of VEGFR-2 and VE-cadherin (Qi et al., 2015b).

Abnormal expression of Wnt2 protein could be seen in CRC cells (Kramer et al., 2017), and overexpression of Wnt2 protein increases the tumor volume and vascular density of CRC xenografts (Jung et al., 2015). Furthermore, Wnt2 levels are associated with the expression of pro-angiogenic proteins, including angiopoietin-2 (Ang-2), interleukin 6 (IL-6), granulocyte colony-stimulating factor (G-CSF), placental growth factor, and vascular markers. Evidence indicates that Wnt2 facilitates angiogenesis by perturbing the physiological equilibrium (Unterleuthner et al., 2019).

### 3.3 Effect of the Wnt/β-catenin signaling pathway on invasion and metastasis of CRC

**FIGURE 4:**
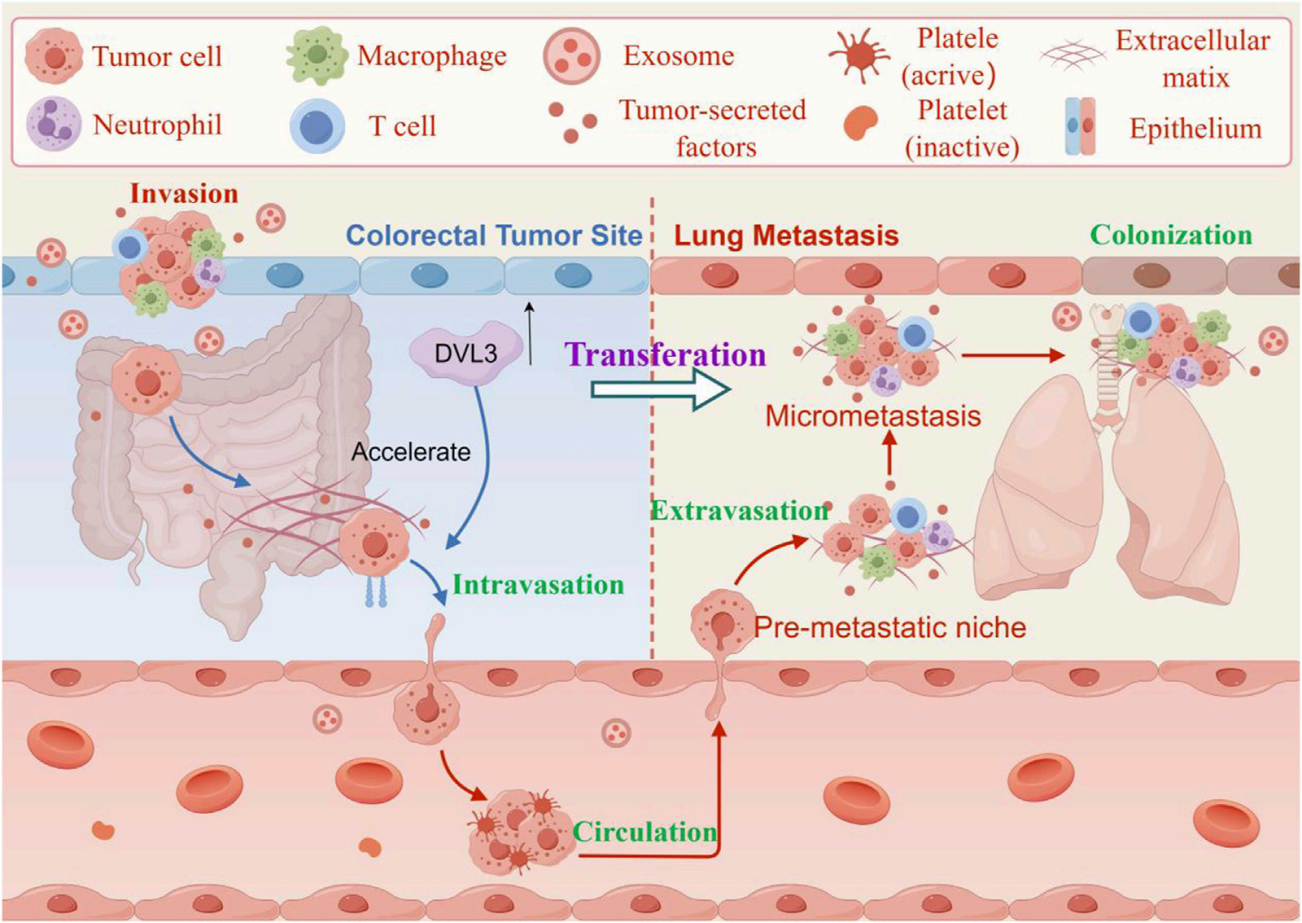
The effect of invasion and metastasis of CRC. The DVL3 recombinant vector is transfected into CRC cells, and overexpression of DVL3 is found to enhance their migration and invasion potential. In contrast, inhibition of DVL3 reduces the migration and invasion capabilities of CRC cells transfected with human influenza virus-8 and human influenza virus-3. These results suggest that DVL3 actively regulates cell metastasis and changes in intestinal cell-like molecules. (The figure was created by Figdraw, https://www.figdraw.com/static/index.html#/).

The gain of cell growth ability is the most basic feature of CRC progression, which will lead to the continuous proliferation of CRC cells, extensive angiogenesis, invasion and metastasis into distant tissues, blood vessels or lymphatic vessels (Hao et al., 2025; Wang et al., 2021b). The metastatic potential of tumors depends largely on the synergistic proteolysis of the basement membrane, the remodeling of the extracellular matrix (ECM) and the epithelial-mesenchymal transition (EMT). EMT serves as a critical determinant in the dissemination of CRC cells during tumor migration and invasion (Banday et al., 2016). It contributes to the progression of various cancers and endows tumor cells with invasive properties and the ability to metastasize to distant organs. When epithelial cancer cells go through the EMT process, they lose tight junctions, cell polarity, and cytoskeletal organization, becoming motile and invasive (Figure 4). Studies have shown that exosomes released by cancer cells play a significant role in the formation of tumor metastasis and chemotherapeutic resistance (Zhou et al., 2018). Metastasis is the leading cause of death in human cancers, including CRC (Wang et al., 2010). In the study, cancer-associated fibroblasts (CAFs) were found to be a key determinant in promoting human CRC growth, invasion, metastasis, and treatment resistance. CAFs transferred exosomes to CRC cells, leading to increased expression of miR-92a-3p in the serum of CRC patients. Meanwhile, the extrachromosomal miR-92a-3p secreted by CAFs could promote cell growth and metastasis, inhibit cell apoptosis and EMT, and mediate chemoresistance (Hu et al., 2019).

Evidence suggested that the overexpression of Rho GTPase Activating Protein 17 (ARHGAP17) inhibit the growth and invasion of CRC cells (including HCT-8 and HCT116) and attenuated lung cancer metastasis by suppressing Wnt/β-catenin signaling (Pan et al., 2018). ARHGAP17 inhibits the invasion and metastasis of CRC cells to the lungs of nude mice and inhibits the occurrence of EMT by inhibiting the Wnt/β-catenin signaling pathway. DVL, a key component of the Wnt/β-catenin signaling pathway, plays a crucial role in stem cell development and adult tissue homeostasis (Shi et al., 2021). DVL3, a member of the DVL family, is overexpressed in primary tumor tissues and multicellular cell lines. The increased expression of DVL3 is negatively correlated with the overall survival rate, disease-free survival rate, and disease-specific survival rate of CRC. The DVL3 recombinant vector was transfected into CRC cells, and proliferation test showed that overexpression of DVL3 increased the migration and invasion potential of HCT-8 cells and SW620 cells. In contrast, inhibition of DVL3 reduced the migration and invasion of SW620 cells transfected with human influenza virus-8 and human influenza virus-3, thus suggesting that DVL3 can positively regulate cell metastasis and intestinal cell-like molecule changes. Meanwhile, the level of DVL3 protein in cancer cells (HT-29, HCT-8, SW480, SW620, HCT116) was higher than that in normal CRC epithelial cells. From the cell cycle point of view, compared with the N0 stage, the DVL3 levels in the N1 and N2 stages of CRC metastasis were higher. DVL3 positively regulated the expression of stem cell surface markers such as cluster of differentiation 44 (CD44) and CD133, and enhanced the expression of stem cell-related transcription factors such as SRY-box transcription factor 2 (SOX2) and c-Myc. The above results indicated that DVL3 promoted cancer cell metastasis and multidrug resistance, and maintained EMT and cancer-stem-like cells (CSLCs) phenotypes through the Wnt/β-catenin/c-Myc/SOX2 axis (Li et al., 2023).

### 3.4 Effect of the Wnt/β-catenin signaling pathway on the progression of CRC

**FIGURE 5:**
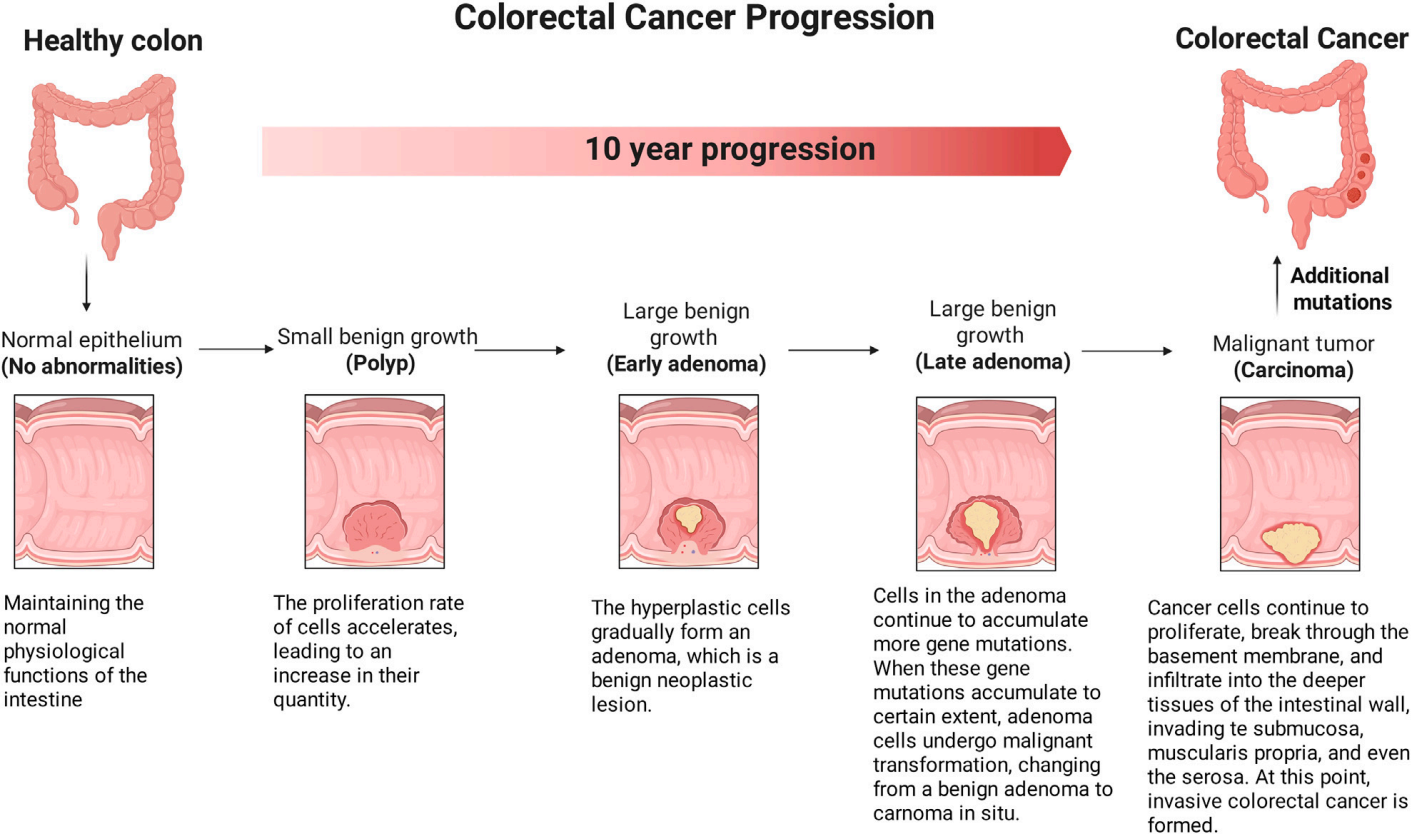
The effect of Wnt/β-catenin signaling pathway on the progression stage of CRC. CRC progression is divided into five stages related to morphological and molecular changes. In the initial stage, the cancer is in the earliest stage and has not yet exceeded the inner mucosa of the colon or rectum. In later stages, the cancer may or may not grow through the walls of the colon or rectum, but will spread to lymph nodes, a distance from the peritoneum (the inner lining of the abdominal cavity), or distant organs (such as the liver or lungs). (The figure was created by Biorender, https://app.biorender.com/).

Monolayer epithelial cells are arranged in the lumen of the mammalian small intestine and colon to form an invagination, called a crypt. Intestinal stem cells (ISCs) drive the massive renewal process within the gut to compensate for the loss of differentiated intestinal epithelial cells at the tips of the crypts (Maartje et al., 2019). As shown in Figure 5, CRC progression is divided into five stages related to morphological and molecular changes. In the initial stage, the cancer remains confined to the inner mucosal layer of the colon or rectum, without extending beyond it, this stage is also known as intramucosal cancer or carcinoma *in situ*. In the second stage, the cancer has grown through the mucosal muscle layer into the submucosa or the muscularis propria, but has not spread to nearby lymphatic nodes or distant sites. In the third stage, the tumor has grown into the outermost layer of the colon or rectum, but has not touched nearby organs, spread to nearby nodes, or metastasized to distant sites (Simon, 2016). In the fourth stage, the cancer has grown from the mucosa to the submucosa, and it may also have reached the muscularis propria, spreading to one or more nearby lymph nodes or to nearby fat. In later stages, the cancer may or may not grow through the walls of the colon or rectum, but will spread to lymph nodes, distant parts of the peritoneum (the inner lining of the abdominal cavity), or distant organs (such as the liver or lungs). It has been reported that the activity of the Wnt/β-catenin signaling pathway is significantly highest specifically at the bottom of the crypts (Farin et al., 2016). Inhibition of the Wnt/β-catenin signal pathway leads to cell proliferation arrest and ISCs deficiency, promoting intestinal epithelial cell ablation (Yu et al., 2024; Zhu et al., 2022), thereby blocking the progression of CRC. During the progression of CRC, the Wnt/β-catenin pathway is activated, and usually accompanied by increased tumor cell proliferation and invasion (Bahrami et al., 2017). Peng et al. discovered in the Gene Expression Omnibus (GEO) that the expression levels of the Wnt/β-catenin signaling pathway target genes, c-Myc and Cyclin D1, in CRC specimens were significantly elevated compared to those in non-tumor specimens. This finding suggests that enhanced modulation of the Wnt/β-catenin signaling pathway and its downstream target genes may facilitate the progression of CRC (Peng et al., 2018).

## 4 Therapeutic applications of pharmacological agents targeting the Wnt/β-catenin signaling pathway in CRC treatment

**FIGURE 6:**
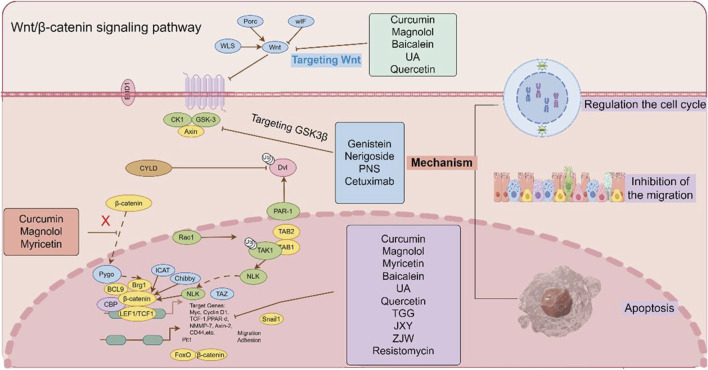
The effect of TCM monomers, formulations and small-molecule inhibitors on the Wnt/β-catenin signaling pathway. TCM monomers, formulations and small-molecule inhibitors have the effect on the Wnt/β-catenin signaling pathway by regulating different targets. (The figure was created by Figdraw, https://www.figdraw.com/static/index.html#/).

The Wnt/β-catenin signaling pathway plays a pivotal role in the pathogenesis and progression of CRC through its regulation of β-catenin stability in the cytoplasm. Consequently, targeting the Wnt/β-catenin pathway has emerged as a promising therapeutic strategy in clinical oncology (Pandey et al., 2023). TCM, characterized by its multi-target and multi-pathway mechanisms, has demonstrated significant anti-cancer efficacy in both preclinical studies and clinical trials, suggesting its potential as an alternative or adjunctive therapeutic approach to conventional chemotherapy in CRC treatment (Tang et al., 2020). As illustrated in Figure 6, the schematic diagram demonstrates the therapeutic mechanisms of TCM monomers, formulations and small-molecule inhibitors in targeting multiple molecular components of the Wnt/β-catenin signaling pathway for the treatment of CRC.

### 4.1 Chinese medicine monomers targeting the Wnt/β-catenin signaling pathway in CRC therapy

Chinese medicine monomers could inhibit β-catenin nclear translocation in CRC cells. Curcumin is a phenolic metabolite extracted from *Curcuma longa L.* (Zingiberaceae; Curcuma longa rhizoma), which has antibacterial, anti-inflammatory, antioxidant and anticancer activities (Xie et al., 2021). It exerts anti-tumor effects by regulating the signal cascade involved in the proliferation, invasion and apoptosis of cancer cells. Curcumin inhibited the nuclear translocation of β-catenin and decresed the expression of its target genes CyclinD1, c-Myc and Axin2 in CRC cells and AOM/DSS model mice, which demonstreted Curcumin play a role in targeting the Wnt/β-catenin signaling pathway (Hao et al., 2021). As a naturally occurring lignan, magnolol is a phenolic metabolite extracted from *Magnolia officinalis Rehd. et Wils.* (Magnoliaceae; Magnolia officinalis bark, root bark, and twig bark), which demonstrates potent antibacterial properties. *In vitro* studies demonstrated that magnolol dose-dependently inhibited the proliferation of SW480 and HCT116 cells. Furthermore, *in vivo* experiments revealed its suppressive effects on tumor growth and invasion in HCT116 xenograft nude mouse models. Mechanistically, these antitumor effects were mediated through the inhibition of β-catenin nuclear translocation and the subsequent disruption of β-catenin/TCF complex binding to its specific DNA binding site in the nucleus (Kang et al., 2012). Myricetin is derived from the leaves, barks and roots of *Myrica rubra* (Myricaceae; Myrica rubra fruit). The metabolite was found to downregulate the Wnt/β-catenin pathway by inhibiting GSK3β activityand reducing the level of cytoplasmic and nuclear β-catenin *in vivo* at 100 mg/kg. In addition, myricetin inhibited the proliferation of intestinal polyps in APC Min^−/+^ mice by modulating β-catenin localization in intestinal adenoma cells, and negatively regulated the expression of the Wnt/β-catenin pathway target gene Cyclin D1 to reduce the number of intestinal polyps (Li et al., 2016) (Table 1).

**TABLE 1:** Chinese medicine monomers targeting the Wnt/β-catenin signaling pathway in CRC.

Metabolites	Biotanical drug source	Type of extract	Experiments	Animal or cell models	Dose range	Pos C	Neg C	Duration	Model	Therapeutic targets	References
Curcumin	Curcuma longa (rhizome)	Polyphenols	AOM + DSS	C57BL/6 male mice	500 mg/kg	--	Water	10 weeks	*In vivo*	β-catenin ↓ Cyclin D1 ↓ c-Myc ↓ Axin2 ↓	Hao et al. (2021)
Magnolol	Magnolia officinalis (bark, root bark, and twig bark)	Lignan	xenograft nude mouse	SW480 cells HCT116 BALB/cA-nu mice	0–50 μg/mL 5 mg/kg	--	DMEM water	24 h 4 weeks	*Invitro* *in vivo*	β-catenin ↓ TCF ↓	Kang et al. (2012)
Myricetin	Myrica rubra (fruits)	Flavonoids	Mice were crossed with wild-type C57BL/6 female mice to generate APCMin/+ mice	C57BL/6 male mice	100 mg/kg	--	CMC-Na 5%	12 weeks	*In vivo*	β-catenin ↓ Cyclin D1 ↓	Li et al. (2016)
Genistein	Glycine max (seeds)	Isoflavones	SW620 xenograft nude mouse	nude mice	0–80 mg/kg	--	Water	7 weeks	*In vivo*	GSK3β ↑MCL1↓APP↓ KDR↓	Zhang et al. (2020)
Baicalein	Scutellaria baicalensis (rhizome)	Flavonoids	xenograft tumor model	HCT116 cells BALB/c nude mice	0–100 μg/mL --	5-Fu	McCoy’s 5A water	48 h 32 days	*In vitro* *in vivo*	GSK3β ↑ E-cadherin ↓	Yang et al. (2020)
UA	Arctostaphylos uva-ursi (leaves)	pentacyclic triterpenoid	xenograft tumor model	NCM460 cells SW620 xenograft tumor model	0–60 μg/mL 15 mg/kg	5-FU	McCoy’s 5A water	48 h 10 weeks	*In vitro* *in vivo*	Cyclin D1↓ β-catenin ↓ GSK3β ↑	Zhao et al. (2023)
Quercetin	Allium cepa (bulb)	Flavonoids	Apc^Min/+^ mice	C57BL/6J mice	17.25 mg/kg	--	water	10.5 weeks	*In vivo*	Cyclin D1 ↓ survivin ↓	Neamtu et al. (2022)
Nerigoside (NG)	Nerium oleander (cardiac glycosides)	Flavonoids	--	HT29 cells SW620 cells	0–800 μg/mL	--	DMEM	48 h	*In vitro*	p-GSK3β ↓ GSK3β ↑ β-catenin ↓	Neamtu et al. (2022)
Sanguinarine	Sanguinaria canadensis (rhizome)	Benzophenanthridine alkaloids	xenograft model	Female nude mice	1.25–5 mg/kg	--	0.5% CMC-Na	7 weeks	*In vivo*	E-cadherin↑ β-catenin ↓	Zhu et al. (2020)
Strychnine	Strychnos nux-vomica (seeds)	Indole alkaloids	DLD1 cells xenograft model	nude mice	5 mg/kg	--	Water	5 weeks	*In vivo*	APC ↓ β-catenin ↓	Ren et al. (2019)
1,4,6-tri-O-galloyl-β-d-glucopyranose (TGG)	Rhus chinensis (gall)	gallotannins	--	HT29 cells	100 ng/mL	--	DMEM	24 h	*In vitro*	β-catenin ↓ Dkk1 ↓ c-Myc ↓ FGF20↓ NKD-1↓ Survivin ↓	Li et al. (2019)
four β HWE	Ginkgo biloba (leaves)	Chalcones	HCT116 xenograft model	HCT116 cells BALB/c-nude mouse	0–1 μg/mL 0–10 mg/kg	--	-- Water	24 h 2 weeks	*In vivo*	β-catenin ↓ AXIN2↓ c-Myc↓ Survivin↓ Cyclin D1↓	Ye et al. (2019)
Columbamine	Coptis chinensis (rhizome)	Isoquinoline alkaloids	HCT116 xenograft model	HCT116 cells BALB/c nude mice	0–40 μg/mL 0–20 mg/kg	--	Water	0–72 h 0–23 days	*In vitro* *in vivo*	MMP2↓ MMP7↓ MMP9↓ N-Cadherin↓ E-cadherin↑	Lei et al. (2019)
Apigenin	*Apium graveolens* (leaves)	flavonoids	--	SW480 cells C57BL/6 mice	0–40 μg/mL --	--	Water	48 h	*In vitro* *in vivo*	Axin2↓ c-Myc↓ Cylin D1↓ Ephb2↓ Ephb3↓	Xu et al. (2016)
Silibinin	Silybum marianum (seeds)	flavonolignans	SW480 xenograft model	athymic (nu/nu) male nude mice	0–200 mg/kg	--	Water	6 weeks	*In vivo*	β-catenin↓ Cyclin D1↓ c-Myc↓ CDK8↓	Kaur et al. (2010)
Chalcone Lonchocarpin	Lonchocarpus spp (resin)	chalcones	AOM + DSS	129SvJxC57BL6 mice	0–100 mg/kg	--	Water	7 weeks	*In vivo*	β-catenin↓ TCF4↓	Predes et al. (2019)

GSK3β regulates the phosphorylation of β-catenin, Chinese medicine monomers could target the GSK3β protein to inhibit the degradation of β-catenin in CRC cells. Genistein is an isoflavone metabolite derived from *Glycine max* (Leguminosae; Glycine max seed). It had been found that genistein could inhibit the Wnt/β-catenin signaling pathway by up-regulating the expression of GSK3β and E-cadherin protein in tumor-transplanted nude mice, thereby inhibiting the metastasis of CRC (Zhang et al., 2020).

Cell cycle-related proteins, including Cyclin A, Cyclin E, Cyclin D1, and CDK2/4, were downregulated in baicalein-induced G1 phase cell cycle arrest. Furthermore, the anti-proliferation and G1 phase arrest effect of baicalein on HCT116 cells were associated with the disruption of the Wnt/β-catenin signaling pathway. Baicalein decresed the expression of Wnt/β-catenin, activated and phosphorylated the degradation complex, then targeted β-catenin for proteasome degradation (Yang et al., 2020). Ursolic acid (UA) is a pentacyclic triterpenoid that has been found in a broad variety of fruits, spices, and medicinal plants. Accumulating evidence has demonstrated that UA exerts potent antitumor effects by significantly inhibiting cellular proliferation, suppressing migratory capacity, and reducing clonogenic potential in SW620 cells. This result was also verified in subcutaneous SW620 xenograft tumor model mice. Mechanistically, UA induces programmed cell death (apoptosis) and triggers cell cycle arrest at the G0/G1 phase through downregulation of the Wnt/β-catenin signaling cascade (Zhao et al., 2023). Quercetin demonstrated dose- and time-dependent cytotoxicity in both SW480 and Clone 26 cells. Following 48-h treatment with quercetin at concentrations ranging from 40 to 80 μM, a significant increase in the percentage of cells arrested at the G2/M phase was observed in both SW480 and Clone 26 cell lines. These findings suggest that the anti-tumor mechanism of quercetin in SW480 cells is mediated through the suppression of Cyclin D1 and survivin expression, coupled with the inhibition of the Wnt/β-catenin signaling pathway (Neamtu et al., 2022). Nerigoside (NG) was isolated from *Nerium oleander Linn.* NG inhibited cell proliferation at low concentrations and induced cell apoptosis at high concentrations. Concurrently, NG significantly impeded cell migration by reversing the EMT and arresting the cell cycle at the G2/M phase (Wen et al., 2019).

EMT serves as a critical determinant in the dissemination of CRC cells during tumor migration and invasion. Chinese medicine monomers could inhibition the EMT process in CRC cells. Cinobufotalin, a bufadienolide isolated from toad venom. It was reported that cinobufotalin inhibited the proliferation of CRC cells significantly. After treatment with cinobufotalin, the invasion and migration of CRC cells could be reduced. Furthermore, cinobufotalin decresed the expression of EMT-related proteins including MMP9, MMP2, N-cadherin and Snail, while increased the expression of E-cadherin protein. And the expression of β-catenin, Wnt3a, c-Myc, Cyclin D1 and MMP7 proteins related to the Wnt/β-catenin signaling pathway were decreased, while the expression of APC protein was increased. In addition, the intestinal fibroma and liver metastases were significantly reduced *in vivo* after cinobufotalin treatment. The above results indicated that cinobufotalin could inhibit the invasion and metastasis of CRC cells through inhibiting the progression of EMT (Wang et al., 2020). Curcumin could inhibit EMT process by regulating the ten-eleven translocation1/Naked cuticle homolog/Wnt (TET1/NKD/Wnt) signaling pathway (Lu et al., 2020). It significantly downregulated the expression of EMT-related genes, including Snail, Twist, N-cadherin, and vimentin, as well as reversed the nuclear translocation of β-catenin. Concurrently, curcumin upregulated the expression of E-cadherin protein, thereby inhibiting the EMT progression in SW480 cells (Chen et al., 2020). Sanguinarine is an alkaloid extracted from Papaveraceae plants such as Macleaya cordata, which exhibits multiple pharmacological effect including anti-inflammation, anti-oxidation, inhibition of cell proliferation and promotion of apoptosis. It was reported that sanguinarine could target the Wnt/β-catenin signaling pathway by inhibiting the EMT process, thereby exerting an anti-metastatic function of CRC (Zhu et al., 2020).

Chinese medicine monomers could enhance the CD8^+^ T cells population in the immune microenvironment. The metabolite Notoginsenoside Ft1 (Ng-Ft1) demonstrated significant inhibitory effects on the formation of subcutaneous tumors in CRC, and it significantly increased the proportion of CD8^+^ T cells in tumor-bearing mice, consequently suppressing tumor growth. Its mechanism may related to Ng-Ft1 selectively targeted the deubitinating enzyme ubiquitin-specific protease 9X (USP9X), disrupting its protective effect on β-catenin, leading to reduce the expression of downstream genes of the Wnt/β-catenin signaling pathway. These finding suggested that Ng-Ft1 may be a promising molecular therapeutic target for CRC by blocking the Wnt/β-catenin signaling pathway and increasing proportion of CD8^+^ T cells within the tumor (Feng et al., 2024).

As a form of programmed cell death, apoptosis serves as an important mechanism in suppressing oncogenesis and CRC development. Chinese medicine monomers could induce the apoptosis in CRC cells. Strychnine, isolated from the seeds of *Strychnos nux vomica L.,* significantly inhibited the growth of DLD1 tumor xenografts in nude mice. Flow cytometry analysis confirmed that its mechanism involves the promotion of cancer cell apoptosis, thereby suppressing CRC progression (Ren et al., 2019). 1,4,6-Tri-O-galloyl-β-d-glucopyranose (TGG), an active metabolite derived from the plant *Sanguisorba officinalis*, effectively inhibited the Wnt/β-catenin signaling pathway. This inhibitory activity was likely mediated through the upregulation of caspase-3, PARP, and the Bax/Bcl-2 ratio, thereby inducing apoptosis in HT29 cells and subsequently suppressing the progression of CRC (Li et al., 2019).

### 4.2 TCM formulations target the Wnt/β-catenin signaling pathway to treat CRC

TCM formulations could protect the intestinal barrier integrity in CRC cells. Prof. Liu proposed the theory “treating tumors from the perspective of membrane theory”, who confirmed that the TCM formulation ChanLinGao (CLG) could inhibit tumor growth, regulate the intestinal microbiota, maintain the integrity of the intestinal mucosal barrier, and suppress intestinal injury. Its mechanism was to protect intestinal tight junction proteins (including occludin, claudin-1 ZO-1, and E-cadherin) from damage, thereby maintaining the integrity of the intestinal mucosal barrier, which in turn inhibited the Wnt/β-catenin signaling pathway and suppressed the initiation and progression of CRC (Tian et al., 2024). Modified Lichong decoction (MLCD) could reduce the abnormal proliferation of intestinal epithelial cells and promote cell apoptosis, thereby inhibit the progression of CRC. The underlying mechanism involved modulation of gut microbiota and disruption of the Wnt/β-catenin signaling pathway. Following MLCD treatment, mice exhibited increased intra-glandular epithelial proliferation in intestinal tissues, reduced focal intestinal tissue ulceration, infiltration of lymphocytes and inflammatory cells into the muscular layer. Additionally, MLCD increased the expression of E-cadherin, decreased the expression of N-cadherin and viment. These findings indicated that MLCD inhibited CRC mainly by suppressing the abnormal proliferation of intestinal epithelial cells, promoting cell apoptosis, and regulating EMT, thereby inhibiting the Wnt/β-catenin signaling pathway (Liu et al., 2024). Pai-Nong-San (PNS) demonstrated significant therapeutic effects in ameliorating AOM/DSS-induced colonic injury in mice, as evidenced by the alleviation of hematochezia, reduction of disease activity index (DAI) scores, increase in colon length, and reversal of pathological alterations. The underlying mechanism primarily involves the modulation of gut microbiota composition, particularly through the regulation of key bacterial populations including Firmicutes, Bacteroidetes, Proteobacteria, and *Lactobacillus*. Further research has revealed that PNS could inhibit the Wnt/β-catenin signaling pathway through downregulating p-GSK3β, β-catenin, c-Myc and GSK3β to suppress CRC development (Zhang et al., 2021) (Table 2).

**TABLE 2:** Chinese medicine formulations targeting the Wnt/β-catenin signaling pathway in CRC.

Biotanical drug	Metabolites	Experiments	Animal or cell models	Dose range	Pos C	Neg C	Duration	Model	Therapeutic targets	References
ChanLinGao (CLG)	Curcuma zedoaria, Rabdosiarubescens, Radix Clematidis, et al.	AOM + DSS	C57BL/6 mice	0–12 mg/kg	--	Water	10 weeks	*In vivo*	Occludin ↑ Claudin-1 ↑ ZO-1 ↑ E-cadherin ↑	Tian et al. (2024)
Modified Lichong decoction (MLCD)	Astragalus membranaceus, Codonopsis pilosula, Atractylodes macrocephala, et al.	AOM + DSS	C57BL/6 mice	0–32.4 g/kg	--	Water	10 weeks	*In vivo*	E-cadherin ↑ N-cadherin ↓ N-vimentin ↓	Liu et al. (2024)
Pai-Nong-San (PNS)	Paeonia lactiflora, Platycodon grandiflorus, Immature bitter orange, et al.	AOM + DSS	BALB/c mice	0–3.2 g/kg	--	Water	11 weeks	*In vivo*	p-GSK3β ↓ β-catenin ↓ c-Myc ↓ GSK3β ↓	Zhang et al. (2021)
Jian Du Xiao Sheng Yin (JXY)	Hedyotis diffusa Willd., Sophora flavescentis aiton, Pleione yunnanensis rolfe, et al.	HCT-15 xenograft nude mouse	BALB/c mice	0–339 mg/kg	--	Water	18 days	*In vivo*	CD133 ↓ DCLK1 ↓ c-Myc ↓ Cyclin D1 ↓	Feng et al. (2025)
Zuo Jin Wan (ZJW)	Coptis chinensis, Tetradium ruticarpum	--	SW403 cells	0–100 μg/mL --	--	fetal bovine serum	0–24 h	*In vitro*	CDK4 ↓ Cyclin D1 ↓ c-Myc ↓ Bcl-2 ↓ p-Bcl-2 (Thr70) ↓ p-Bcl-2 (Ser56) ↓	Pan et al. (2017)
Weichang’an (WCA)	Dolomiaea souliei, Santalum album, Rheum palmatum	--	HCT116 cells	0–1.6 mg/mL	--	DMEM	0–24 h	*In vitro*	MMP7 ↓ MMP9 ↓ ZEB1 ↓ β-catenin ↓	Tao et al. (2019)

The TCM formulation Jian Du Xiao Sheng Yin (JXY) has been showed to exert anti-tumor effect by inhibiting cancer cell and angiogenesis. JXY extract inhibited the expression and transcriptional activity of β-catenin protein, while also suppressing target proteins of the Wnt/β-catenin pathway, such as c-Myc and Cyclin D (Feng et al., 2025). The Zuo Jin Wan (ZJW) extract could increase the expression of 5-hydroxytryptamine receptor D (5-HTR1D) and axin-1 and decrease the expression of LEF1, TCF4, MMP2, MMP7, ICAM-1, and CXCR4 to inhibit the 5-HTR1D-Wnt/β-catenin signaling pathway in SW480 cells (Pan et al., 2017). The Chinese botanical formulation Weichang’an (WCA) inhibited Wnt/β-catenin signaling pathway by upregulating rho GTPase activating protein 25 (ARHGAP25) expression. WCA (0.4 mg/mL) downregulated the expression of MMP7, MMP9, zinc finger E-box binding homeobox 1 (ZEB1), and β-catenin to anti-CRC migratory and invasive in HCT116 cells (Tao et al., 2019).

### 4.3 Small-molecule inhibitors targeting the Wnt/β-catenin signaling pathway to treat CRC

Mei et al. found that the scratch wound healing rate and cells pass through the transwell membrane of CRC cells in the protocadherin gamma subfamily A, 9 (PCDHGA9) knockdown group was greater, while the opposite results were observed in the PCDHGA9 overexpression group. Among the EMT-related E-cadherin protein level in the PCDHGA9 knockdown group was significantly reduced, while the expression of N-cadherin, imentin, and Snail proteins was significantly increased, and the PCDHGA9 overexpression group showed the opposite trend (Mei et al., 2024). Resistomycin is a natural antibiotic with potent anti-tumor activity. It could inhibit the proliferation of SW480 and HCT116 cells in a dose and time-dependent manner. Further studies found that resistomycin could promote the expression of caspase3 and reduce the expression of Bcl-2. Immunofluorescence and flow cytometry analyses confirmed that resistomycin could induce dose-dependent cell apoptosis in both HCT116 and SW480 cell lines. To further reveal the mechanism of resistomycin-induced apoptosis in CRC cells, CRC cells were treated with lithium chloride (APC/Axin/GSK3β complex inhibitor) alone and in combination with resistomycin. It was found that lithium chloride reversed the total, cytoplasmic and nuclear protein expression of β-catenin and the total protein expression of c-Myc protein in the Wnt/β-catenin signaling pathway by resistomycin. In summary, resistomycin-induced apoptosis of CRC cells was achieved by targeting the Wnt/β-catenin signaling pathway (Zhu et al., 2023) (Table 3).

**TABLE 3:** Small-molecule inhibitors targeting the Wnt/β-catenin signaling pathway in CRC.

Small-molecule inhibitors	Experiments	Animal or cell models	Dose range	Pos C	Neg C	Duration	Model	Therapeutic targets	References
PCDHGA9	--	HCT116	--	--	--	0–48 h	*In vitro*	CXCR4 ↓ β-catenin ↓	Mei et al. (2024)
Resistomycin	--	HCT-116 SW620 cells	0–1 μg/mL	--	DMEM	0–72 h	*In vitro*	β-catenin ↓ TCF4 ↓ GSK3β↓ c-Myc ↓ caspase-3 ↑ Bax ↑ Bcl-2↓	Zhu et al. (2023)
Bevacizumab	MDA-MB-231/MDA-MB-468 xenografts	nude mice	0–25 mg/kg	--	water	--	*In vivo*	N-cadherin ↓ Vimentin ↓ ZEB ↓ Snail ↓ Slug ↓ E-cadherin↑	Xie et al. (2019)
Cetuximab	--	--	--	--	--	--	*--*	APC ↓ TP53 ↓	Qiu et al. (2021)
C-1	HCT116 xenograft models	NSG mice	0–4 mg/kg	--	water	20 days	*In vivo*	β-catenin ↓ Bcl-9 ↓	Tanton et al. (2022)
PTK7	--	HCT116 SW480 cells	0–25 μg/mL	--	--	0–24 h	*In vitro*	APC ↓ β-catenin ↓	Anier et al. (2022)

Angiogenesis is a complex process regulated by various pro-angiogenic factors and anti-angiogenic factors, which is essential for tissue growth and development. VEGF is a key regulator of angiogenesis in physiological and pathological processes, such as cancer development (Battaglin et al., 2018). Bevacizumab is a recombinant humanized monoclonal antibody that inhibits angiogenesis by inhibiting VEGF, thereby promoting tumor cell invasion and metastasis. This result suggested that bevacizumab induced EMT while exerting its tumor-inhibitory effects through the Wnt/β-catenin signaling pathway (Xie et al., 2019).

Epidermal growth factor receptor (EGFR) plays a key role in tumor evolution, proliferation and immune escape, and is one of the most important targets for the biotherapy of CRC. Tumor-promoting events mediated by EREG are generated through autocrine and paracrine signaling loops, these loops were initiated by tumor epithelial cells, fibroblasts, and macrophages within the TME (Cheng et al., 2021). Due to mutations in the Wnt/β-catenin signaling pathway, CRC tumors are highly metastatic and invasive, which leads to the rapid recurrence of metastatic tumors in a short period after resection. Research had found that metastatic tumors with activating mutations in the Wnt/β-catenin signaling pathway would recur soon after surgery. The combination of cetuximab and chemotherapy could significantly reduced the volume and number of metastatic tumors (Qiu et al., 2021). This finding declared that cetuximab treat CRC by mediating the EGFR through the Wnt/β-catenin signaling pathway.

It was reported that C-1 (small-molecule inhibitors of the interaction between β-catenin and its coactivator Bcl-9) could bound to β-catenin and downregulated expression of the Wnt/β-catenin, disrupted cholesterol homeostasis, and significantly reduced the proliferation of CRC cell lines and tumor growth in a xenograft mouse model of CRC (Tanton et al., 2022). Pseudokinase PTK7 intervenes in the regulation of the Wnt/β-catenin pathway signaling, so the inhibition of the PTK7/β-catenin interaction could represent a new therapeutic strategy to inhibit CRC cell growth (Anier et al., 2022).

## 5 Challenges and future perspectives

During the progression of CRC, dysregulation of the Wnt/β-catenin signaling pathway promotes tumor invasion and metastasis, accelerating disease development. TCM has demonstrated significant efficacy in the clinical management of tumor development and progression (Sun et al., 2024). By targeting the Wnt/β-catenin signaling pathway, TCM can inhibit the proliferation and metastasis of tumor cells, induce apoptosis, and reverse resistance to chemotherapy (Chen et al., 2023). Related basic and clinical studies have further elucidated the mechanisms of TCM in the precise prevention and treatment of CRC. Several TCM metabolites, including baicalein, UA, quercetin, TCM formulations such as CLG, JXY, ZJW, and small molecule inhibitors like PCDHGA9, C-1, PTK7, which have demonstrated beneficial effects in CRC models. These findings suggest that herbal formulations and metastasis derived from TCM may exert their anti-CRC potential by modulating the Wnt/β-catenin signaling pathway, thereby inhibiting the nuclear translocation of β-catenin, promoting apoptosis in CRC cells, suppressing angiogenesis, attenuating the EMT process, and arresting cell cycle progression. These results provide compelling evidence supporting the efficacy of TCM-based herbal formulations and metastasis in CRC treatment, highlighting the critical role of targeting the Wnt/β-catenin signaling pathway. Despite significant progress in fundamental research on TCM for CRC, the inherent complexity of TCM components, along with unidentified factors and intricate *in vivo* modifications, renders the current findings largely speculative. Further in-depth investigations are imperative to elucidate the specific active components and molecular mechanisms underlying TCM interventions in CRC. Such efforts will enhance our understanding of their therapeutic effects and provide a solid foundation for guiding clinical applications. Below, we outline the limitations in research design, existing challenges, and future research directions for TCM-based CRC treatment.

Although TCM formulations and their metabolites demonstrate promising therapeutic potential in regulating the Wnt/β-catenin signaling pathway for the treatment of CRC, several critical issues remain unresolved. Firstly, there is a significant lack of clinical research data, making it difficult to validate their clinical efficacy. Secondly, in existing basic studies, some TCM have only been investigated *in vitro* without *in vivo* validation, and common limitations include insufficient use of positive control drugs, as well as unclear composition and ratios of TCM formulations. To overcome these research bottlenecks, future studies should conduct more systematic *in vivo* experiments to simulate the complex physiological microenvironment, while designing more rigorous experimental protocols to elucidate the precise mechanisms by which TCM target the Wnt/β-catenin signaling pathway in CRC treatment. Additionally, comprehensive evaluation of their efficacy and safety is essential. Furthermore, efforts should be directed toward optimizing the preparation processes and clinical application standards of TCM formulations. By integrating modern analytical approaches such as metabolomics and network pharmacology, higher-quality and more in-depth mechanistic studies can be promoted, thereby providing more reliable evidence-based support for the clinical application of TCM in CRC treatment.

TCM modulate the Wnt/β-catenin signaling pathway through direct or indirect mechanisms targeting multiple nodal points. This multilevel regulation involves: (i) activation of upstream receptors, (ii) modulation of key kinases, and (iii) alteration of downstream effector proteins. For example, UA induces programmed cell death (apoptosis) and triggers cell cycle arrest at the G0/G1 phase through downregulation of the Wnt/β-catenin signaling cascade (Zhao et al., 2023). Ng-Ft1 may be a promising molecular therapeutic target for CRC by blocking the Wnt/β-catenin signaling pathway and increasing proportion of CD8^+^ T cells within the tumor (Feng et al., 2024). MLCD effectively suppresses CRC progression by attenuating abnormal intestinal epithelial cell proliferation and promoting apoptosis. This inhibitory effect is mediated through dual mechanisms: (1) modulation of gut microbiota composition, and (2) disruption of both the Wnt/β-catenin signaling pathway and EMT processes (Liu et al., 2024). JXY extract inhibited the expression and transcriptional activity of β-catenin protein, while also suppressing target proteins of the Wnt/β-catenin pathway, such as c-Myc and Cyclin D (Feng et al., 2025). Although current studies have identified how certain TCM modulate the Wnt/β-catenin pathway, the direct molecular targets and precise regulatory mechanisms of specific bioactive constituents remain elusive. Further mechanistic investigations are warranted to elucidate these critical aspects.

Beyond the Wnt/β-catenin signaling pathway, accumulating evidence indicates that formulations and bioactive metabolites derived from TCM can effectively ameliorate CRC pathogenesis through multi-target modulation of critical signaling pathways. These include the PI3K/Akt, NF-κB, MAPK, epidermal growth factor receptors, p53, TGF-β, mTOR, Hedgehog, and immunomodulatory signaling pathways (Chen et al., 2023). For example, Sijunzi Decoction (SJZD) could promote apoptosis and autophagy of CRC cells through PI3K/Akt/mTOR pathway (Shang et al., 2023). Allicin improves the sensitivity of X-ray radiotherapy in CRC, which mechanism may be associated with inhibition of NF-κB signaling pathway (Huang et al., 2020). This polypharmacological action underscores TCM’s unique therapeutic advantage in simultaneously targeting multiple pathological mechanisms underlying CRC progression. However, systematic investigations are still required to fully elucidate the pathway crosstalk and network pharmacology mechanisms of these TCM-based interventions.

TCM, an integral component of China’s distinguished ancestral cultural heritage with a history spanning over 3,000 years, comprises unique botanical formulations derived from diverse medicinal plants. These formulations have been extensively utilized in clinical practice for managing various diseases. Contemporary scientific investigations have increasingly highlighted the potential of TCM in the effective prevention and treatment of CRC. Ongoing research continues to explore the therapeutic and preventive efficacy of classical TCM formulations. Notably, a randomized, double-blind, placebo-controlled clinical trial demonstrated that the Jianpi Lishi Jiedu granules (JLJ) treatment group exhibited a significantly lower recurrence rate of 20%, compared to 35% in the control group (Wu et al., 2022). A comprehensive meta-analysis investigating the therapeutic effects of Platycodon grandiflorus and Astragalus membranaceus demonstrated their efficacy in reducing postoperative wound infections and enhancing recovery outcomes following CRC surgery. The analysis revealed a statistically significant reduction in hospital length of stay (LOS) in the Platycodon grandiflorus and Astragalus membranaceus treatment group compared to the control group, with a mean difference of 1.2 days (95% CI: 0.8–1.6 days, p < 0.01) (Chen et al., 2024). These findings underscore the potential of integrating TCM into postoperative care protocols, suggesting its capacity to improve patient outcomes and contribute to enhanced antibiotic stewardship in surgical settings.

Currently, drugs directly targeting the Wnt/β-catenin pathway remain in the developmental phase. In this context, investigating the regulatory effects of active components from TCM and their metabolites on the Wnt/β-catenin pathway may provide critical insights for developing novel targeted therapeutics. TCM offers unique advantages through its multi-component system that exerts synergistic effects by simultaneously modulating multiple critical nodes of the Wnt/β-catenin pathway, including both upstream regulators and downstream effector molecules. Given the complex pathogenesis of CRC, the development of multi-target agents may be essential to achieve optimal therapeutic outcomes.

Clinical studies have demonstrated that TCM formulations and their bioactive constituents exhibit distinctive advantages in CRC treatment, including reduced adverse effects, convenient administration routes, and lower treatment costs. However, current clinical evaluation systems present several limitations: (1) therapeutic assessments predominantly rely on symptomatic improvement rather than objective biomarker validation; (2) standardized treatment protocols are lacking; and (3) potential toxicity may emerge with prolonged use of certain compounds. Therefore, there is an urgent need to conduct large-scale, multicenter, high-quality clinical trials to rigorously evaluate the efficacy and safety of TCM in CRC management.
